# MeCP2-E1 isoform is a dynamically expressed, weakly DNA-bound protein with different protein and DNA interactions compared to MeCP2-E2

**DOI:** 10.1186/s13072-019-0298-1

**Published:** 2019-10-10

**Authors:** Alexia Martínez de Paz, Leila Khajavi, Hélène Martin, Rafael Claveria-Gimeno, Susanne Tom Dieck, Manjinder S. Cheema, Jose V. Sanchez-Mut, Malgorzata M. Moksa, Annaick Carles, Nick I. Brodie, Taimoor I. Sheikh, Melissa E. Freeman, Evgeniy V. Petrotchenko, Christoph H. Borchers, Erin M. Schuman, Matthias Zytnicki, Adrian Velazquez-Campoy, Olga Abian, Martin Hirst, Manel Esteller, John B. Vincent, Cécile E. Malnou, Juan Ausió

**Affiliations:** 1 Department of Biochemistry and Microbiology, University of Victoria, Petch Building 260, Victoria, BC V8W 3P6 Canada; 2 Unité de Mathématiques et Informatique Appliquées, Toulouse INRA, Auzeville, BP 52627, 31326 Castanet-Tolosan Cedex, France; 3 Centre de Physiopathologie de Toulouse Purpan, INSERM, UMR 1043, CNRS, UMR 5282, Université Toulouse III Paul Sabatier, Toulouse, France; 4 Institute of Biocomputation and Physics of Complex Systems (BIFI), Joint Units IQFR-CSIC-BIFI and GBsC-CSC-BIFI, Universidad de Zaragoza, 50018 Saragossa, Spain; 5 Instituto Aragonés de Ciencias de la Salud (IACS), 50009 Saragossa, Spain; 6 Aragon Institute for Health Research (IIS Aragon), 50009 Saragossa, Spain; 7 Synaptic Plasticity Department, Max-Planck-Institute for Brain Research, Frankfurt/Main, Germany; 8 School of Life Sciences, Brain Mind Institute, École Polytechnique Fédérale de Lausanne, 1015 Lausanne, Switzerland; 9 Michael Smith Laboratories, University of British Columbia, Vancouver, BC V6T 1Z4 Canada; 10 Department of Microbiology and Immunology, University of British Columbia, Vancouver, BC V6T 1Z4 Canada; 11 University of Victoria-Genome British Columbia Proteomics Centre, Vancouver Island Technology Park, #3101-4464 Markham Street, Victoria, BC V8Z7X8 Canada; 12 Molecular Neuropsychiatry & Development (MiND) Lab, Campbell Family Mental Health Research Institute, Centre for Addiction and Mental Health, Toronto, ON M5T 1R8 Canada; 13 Institute of Medical Science, University of Toronto, Toronto, ON M5S 1A8 Canada; 14 Department of Biochemistry and Microbiology, University of Victoria, Room 270d, Petch Building, 3800 Finnerty Road, Victoria, BC V8P 5C2 Canada; 15 Gerald Bronfman Department of Oncology, Jewish General Hospital, Suite 720, 5100 de Maisonneuve Boulevard West, Montreal, QC H4A 3T2 Canada; 16 Proteomics Centre, Segal Cancer Centre, Lady Davis Institute, Jewish General Hospital, McGill University, 3755 Côte-Sainte-Catherine Road, Montreal, QC H3T 1E2 Canada; 17 Department of Biochemistry and Molecular and Cell Biology, Universidad de Zaragoza, 50009 Saragossa, Spain; 18 Fundación ARAID, Government of Aragon, 50018 Saragossa, Spain; 19 Biomedical Reseach Networking Centre for Liver and Digestive Diseases (CIBERehd), Madrid, Spain; 20 Michael Smith Genome Sciences Centre, BC Cancer Agency, Vancouver, BC V5Z 1L3 Canada; 21 Cancer Epigenetics and Biology Program (PEBC), Bellvitge Biomedical Research Institute (IDIBELL), Avinguda Gran Vía de L’Hospitalet 199-203. L’Hospitalet de Llobregat, Barcelona, Catalonia Spain; 22 Physiological Sciences Department, School of Medicine and Health Sciences, University of Barcelona (UB), Catalonia, Spain; 23 Institució Catalana de Recerca I Estudis Avançats (ICREA), Barcelona, Catalonia Spain; 24 Department of Psychiatry, University of Toronto, Toronto, ON M5T 1R8 Canada; 25 Present Address: Department of Pathology and Laboratory Medicine, Weill Cornell Medical College, Cornell University, New York, NY 10065 USA

**Keywords:** MeCP2, Isoforms, Chromatin, Rett syndrome

## Abstract

**Background:**

MeCP2—a chromatin-binding protein associated with Rett syndrome—has two main isoforms, MeCP2-E1 and MeCP2-E2, differing in a few N-terminal amino acid residues. Previous studies have shown brain region-specific expression of these isoforms which, in addition to their different cellular localization and differential expression during brain development, suggest that they may also have non-overlapping molecular mechanisms. However, differential functions of MeCP2-E1 and E2 remain largely unexplored.

**Results:**

Here, we show that the N-terminal domains (NTD) of MeCP2-E1 and E2 modulate the ability of the methyl-binding domain (MBD) to interact with DNA as well as influencing the turn-over rates, binding dynamics, response to neuronal depolarization, and circadian oscillations of the two isoforms. Our proteomics data indicate that both isoforms exhibit unique interacting protein partners. Moreover, genome-wide analysis using ChIP-seq provide evidence for a shared as well as a specific regulation of different sets of genes.

**Conclusions:**

Our study supports the idea that Rett syndrome might arise from simultaneous impairment of cellular processes involving non-overlapping functions of MECP2 isoforms. For instance, MeCP2-E1 mutations might impact stimuli-dependent chromatin regulation, while MeCP2-E2 mutations could result in aberrant ribosomal expression. Overall, our findings provide insight into the functional complexity of MeCP2 by dissecting differential aspects of its two isoforms.

## Background

Methyl CpG-binding protein 2 (MeCP2) was first identified through its ability to bind methylated DNA [1]. Mutations in the *MECP2* gene were later associated with Rett syndrome (RTT; OMIM 312750), a severe neurological disorder that is among the most common causes of intellectual disability in females [2].

**Fig. 1:**
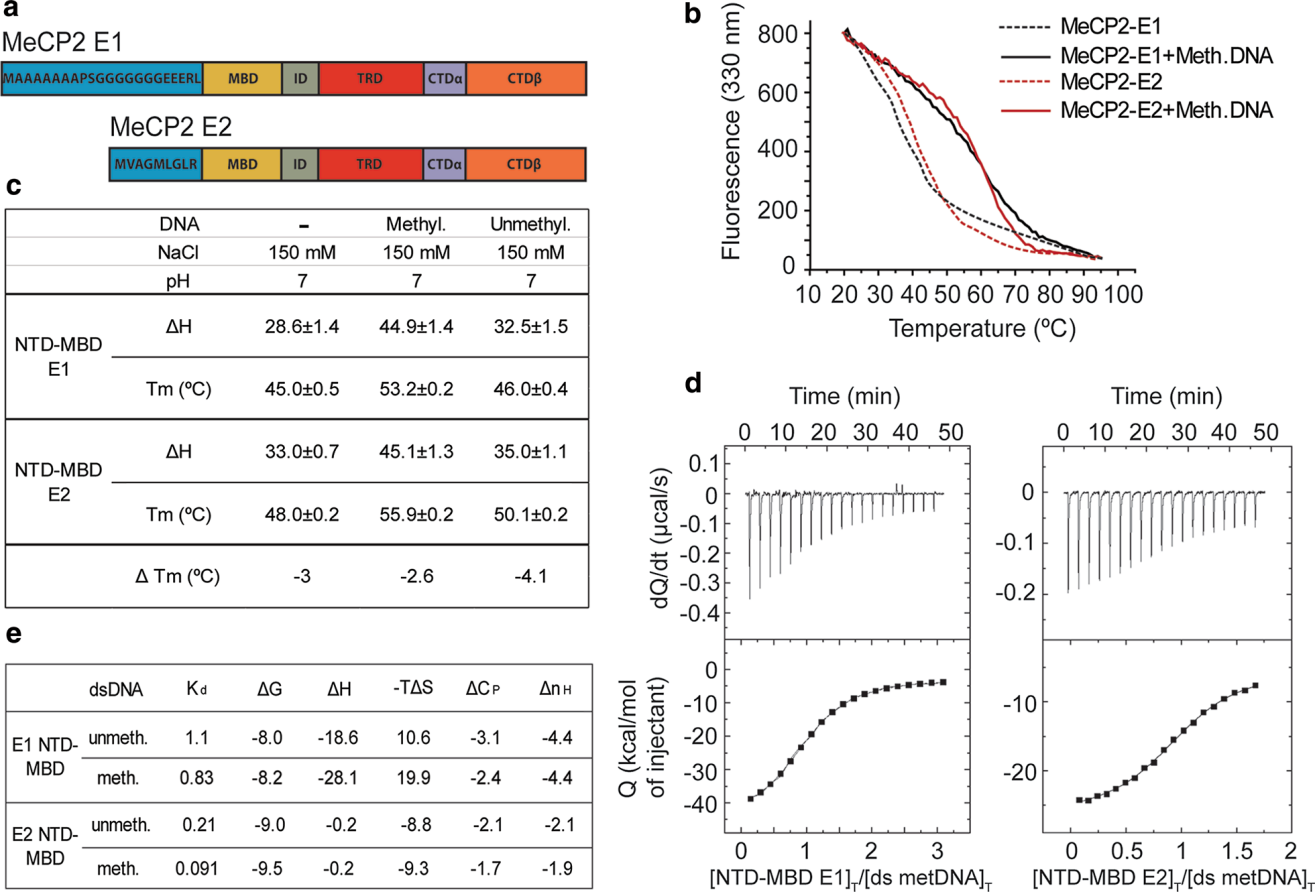
Biophysical characterization of the MeCP2-E1 and E2 NTD-MBD domain interaction with DNA. **a** Schematic representation of the MeCP2-E1 and E2 isoforms depicting the unique NTD amino acid sequences and shared domains. **b** Fluorescence thermal denaturation curves for E1 and E2 NTD-MBD protein fragments in the presence of unmethylated and mCpG-dsDNA. Unfolding traces were fitted considering a two-state unfolding model. **c** Unfolding stability parameters obtained from thermal denaturations followed by intrinsic tryptophan fluorescence. **d** Calorimetric titrations of E1 and E2 NTD-MBD proteins interacting with dsDNA plots show the thermograms (thermal power as a function of time) and the binding isotherms (normalized heats as a function of the dsDNA/protein molar ratio). **e** Buffer-independent dsDNA binding parameters (K_d_, dissociation constant; Δ*G*: Gibbs free energy of interaction; Δ*H*: enthalpy of interaction; −*T*Δ*S*: entropic contribution of interaction; Δ*C*_*P*_: heat capacity of interaction; *n*_*H*_: number of protons exchanged upon complex formation) obtained from calorimetric titrations at pH 7

**Fig. 2:**
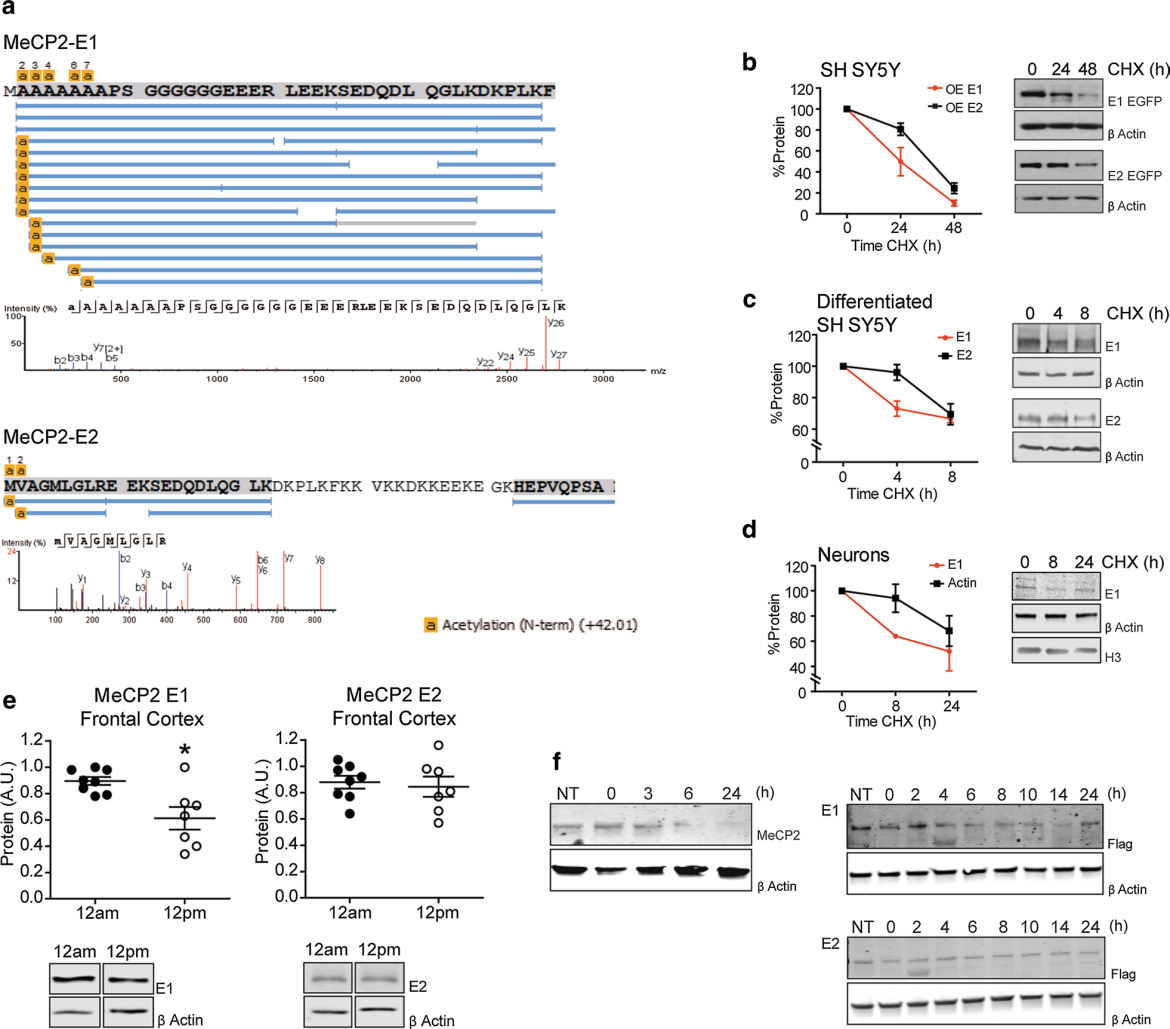
Isoforms N-terminal processing, turn-over rates, and dynamics. **a** Mass spectrometry sequencing of the N-terminal end of the MeCP2 protein (in vitro). N-terminal peptide coverage alignment chart and high-resolution mass spectra showing *N*-methionine excision (NME) and *N*-acetylation (NA) of the N-termini of MeCP2-E1 and MeCP2-E2. NA (+42 Da) of N-terminus amino acid is shown highlighted in yellow. **b**–**d** Cycloheximide-chase assays of the E1 and E2 MeCP2 isoforms. Densitometries and representative western blots performed after cycloheximide treatments of **b** SH-SY5Y cells overexpressing (OE) E1 and E2 isoforms fused to GFP, **c** differentiated SH-SY5Y, and **d** rat cultured cortical neurons with detection of endogenous E1 and E2 isoforms. **e** Densitometric analysis and representative western blots showing endogenous E1 and E2 levels in frontal cortices of mice euthanized at 12 a.m. and 12 p.m. **f** KCL depolarization and representative Western blots analysis of total endogenous MeCP2 of cultured cortical neurons and E1 and E2 of transfected cultured cortical neurons overexpressing Flag-MeCP2-E1 or E2. Represented data are mean ± S.E.M. (*n* = 7–8). * *P *< 0.05 two-tailed Mann–Whitney test. MeCP2 levels were normalized using β actin and/or histone H3

*MeCP2* gene has four exons than can be alternatively spliced to produce two transcripts. The transcript skipping exon 2 has translation initiation in exon 1 and encodes MeCP2-E1. This isoform is slightly longer (498 amino acids in humans) and has 21 unique N-terminal amino acids. When exon 2 is included in the transcript, translation initiates in exon 2 to give rise to MeCP2-E2, a shorter variant (486 amino acids in humans) with 9 unique N-terminal amino acids [3, 4]. The remaining sequence is identical for both isoforms (Figs. 1a and 2a).

MeCP2-E1 is likely the ancestral form of the protein, as orthologues are present across vertebrate evolution, whereas orthologous sequences of the exon 2 coding region have only been found in mammalian genomes [5]. Although splicing variants often encode proteins with different functions, in the case of MeCP2-E1 and E2 isoforms, this remains still controversial [6, 7]. The presence of a polyalanine tract followed by a polyglycine tract in E1 N-terminal domain (NTD) could be an indication of a potential functional difference [8]. In this regard, polyalanine domains within various protein families are thought to have a convergent origin, suggesting that a specific function for these tracts has been selected by evolutionary pressure [9]. The existence of non-overlapping functions of the E1 and E2 isoforms is supported by a difference in their relative abundance during development and in diverse regions of the brain [10, 11]. Moreover, Rett syndrome-causing mutations described so far involve solely the E1 isoform, and isoform-specific mouse knockouts show Rett-related phenotypes for E1 knockout but not for E2, suggesting that E2 does not functionally compensate for the lack of E1 [12, 13]. However, the high degree of structural similarity between MeCP2 isoforms points towards a high extent of functional overlapping, and some findings reinforce this idea. For instance, E2 expressed at levels comparable to those of E1 was reported to prevent key Rett-like phenotypes in mice models of Rett syndrome, indicating that part of the difference between isoforms could simply be related to the disparity in temporospatial expression and protein levels [7].

Given the poorly understood nature of the structural and functional differences between E1 and E2 isoforms, we decided to investigate this further. Our study comprehensively describes for the first time differences between MeCP2 isoforms, using various complementary biophysical, biochemical, and genomic approaches. This work provides a detailed framework for the further understanding of the many fold functional aspects of MeCP2, thus shedding light onto the pathophysiology of Rett syndrome and other neurological disorders.

## Results

### Biophysical characterization of MeCP2 isoforms N-terminal domains

As mentioned in the introduction, the different functionality of the MeCP2 isoforms has long remained controversial. However, there are many indirect hints to suggest otherwise, including a different pattern of expression during mouse brain development (Additional file 1: Fig. S1 A) [11] and various evidences previously mentioned. Interestingly, the two MeCP2 isoforms differ only in their N-terminal domain (NTD) (Fig. 1a), which has been previously described to lack any DNA-specific binding structure, but has the ability to stabilize the neighboring methyl-binding domain (MBD) and its binding to methylated DNA [14, 15]. A partial folding of this unstructured region might contribute to the interaction with double-stranded DNA (dsDNA), thus having a differential impact on E1 and E2 binding properties. Therefore, we decided to compare the different biophysical properties of E1 and E2 NTDs. Constructs consisting of the E1 or E2 specific NTD followed by the MBD were analyzed as previously described [15]. Thermal unfolding studies of E1/NTD-MBD and E2/NTD-MBD (Fig. 1b) indicate that E1 isoform shows a slightly lower mid-transition temperature (temperature at which 50% of the protein is unfolded, *T*_m_) in all situations considered according to the two-state unfolding model (Fig. 1c; Additional file 1: Fig. S1B), showing a slightly lower structural stability.

Following the same trend, E1 isoform also shows a diminished unfolding enthalpy (Δ*H*) (Fig. 1c), indicating a lower cooperativity in the thermal unfolding, suggesting that amino acid residues located at the NTD might be important for the stability of the folded regions located in the MBD. The nature of protein–DNA interactions was further assessed by determining their thermodynamic profile with isothermal titration calorimetry (ITC), considering a single binding site model [15] (Fig. 1d, e). Results show that compared to E2, E1 exhibits ninefold lower binding affinity (higher dissociation constant, *K*_d_) for methylated dsDNA and fivefold lower binding affinity for unmethylated dsDNA, thus resulting in E1 isoform having a slightly lower discrimination capability for methylated/unmethylated dsDNA (Fig. 1e). Strikingly, the main intermolecular DNA-binding driving forces for the two isoforms are of different nature, displaying opposed thermodynamic binding profiles: dsDNA interaction with E1 is enthalpically driven and with E2 is entropically driven; thus, while E1 interacts with favorable binding enthalpy (Δ*H*) and unfavorable binding entropy (−*T*Δ*S*), E2 interacts with negligible binding enthalpy and favorable binding entropy (Fig. 1e). Therefore, the interaction of E1 isoform with dsDNA is mainly driven by specific interactions between the protein and the dsDNA (i.e., hydrogen bonds and electrostatic interactions), and the interaction of E2 isoform with dsDNA is mainly driven by unspecific interactions (i.e., hydrophobic desolvation and steric arrangements). In addition, E1 isoform exhibits a larger binding heat capacity (Δ*C*_P_) and the formation of its complex with dsDNA releases a larger number of protons (*n*_H_). Overall, these observations indicate that the amino acid residues at the N-terminal regions of E1 and E2 NTDs have significant influence not only on protein stability, but also on the interaction with the dsDNA: E1 is slightly less stable and exhibits lower affinity for dsDNA than E2 isoform. Fluorescence recovery after photobleaching (FRAP) data for the two isoforms supports this, with E1 having a more rapid recovery trajectory than E2, suggesting looser binding, although t-half and mobile fractions were not significantly different (Additional file 1: Fig. S1C). These properties could also be reflecting a differential ability of MeCP2 isoforms to interact with other molecules, different turn-over rate, intracellular trafficking, or susceptibility to undergo post-translational modifications.

### Higher MeCP2-E1 protein turn-over in neuronal systems reflects its involvement in dynamic processes

MeCP2 is an intrinsically disordered protein (IDP) [16], thus highly susceptible to proteolytic degradation. The lower affinity of the E1 NTD-MBD region for DNA or its lower folding stability might reflect a higher presence in solution or the occurrence of a larger exposed surface to be targeted for proteasomal degradation [17]. Therefore, we decided to compare the half-lives of the two MeCP2 isoforms in different neuronal systems. The end-terminal amino acid has great impact in protein degradation [18], and thus, we first assessed the N-terminal processing of both proteins. The NTDs were expressed in HEK293T cells, and purified and analyzed by mass spectrometry (MS). Our previous MS sequencing of the N-terminal tael of MeCP2-E1 [5] showed no peptides with N-terminal methionine (NM), indicating complete NM excision (NME) at the first residue (P1) position (Fig. 2a Top panel). Acetylation of the initial alanine residue (P′1) after NME was observed.

In addition, we observed some peptide reads with alanine 1, or alanine 1 and 2, or alanine 1–4, or 1–5 excised and acetylation of the subsequent alanine. For MeCP2-E2, on the other hand, we found reads in which N-terminal methionine (P1 position) is retained and acetylated and few peptide reads with NME and acetylation of the penultimate valine (P’1) (Fig. 2a Bottom panel). All post-translational modifications (PTMs) reported received Ascores of 1000. The complete methionine excision and the presence of alanine as first residues in E1 support a faster turn-over rate of this isoform, compared to the E2 bearing a methionine or valine as N-terminal amino acids [19]. We then decided to test this possibility by performing cycloheximide (CHX) chase assays and Western blot (WB) in three different contexts: undifferentiated SH-SY5Y neuroblastoma cells transfected with E1- and E2-EGFP (enhanced green fluorescent protein) fusion proteins (Fig. 2b), differentiated SH-SY5Y (Fig. 2c), Additional file 2: Fig. S2A), and DIV7 rat cortical neurons (Fig. 2d). These experiments consistently showed a tendency of E1 to be degraded faster than E2, both at the endogenous level and when the isoforms were overexpressed in cell cultures (detected using specific in house-made antibodies, Additional file 2: Fig. S2B). Our results prompted us to investigate if MeCP2 isoforms would show differential behavior in two highly dynamic neuronal settings, the circadian cycle and neuronal activation. First, we took advantage of the system which we reported previously displaying total MeCP2 24 h oscillations [20]. Analysis of frontal cortices obtained at 12 a.m. and 12 p.m. shows a noticeable 30% reduction of E1 protein level at 12 p.m., while E2 levels remain similar at these two times (Fig. 2e). The second scenario involving MeCP2 dynamics was neuronal activation after KCl exposure. Protein levels were measured at different time points after depolarization (55 mM KCl). The inability of the MeCP2-E2 antibody to detect this isoform in rat neurons prompted us to first determine the endogenous levels of total MeCP2 after treatment. Due to the high E1 abundance compared to E2, this mostly corresponds to the E1 isoform. Next, we assessed the two isoforms’ dynamics by transfecting cultured rat neurons with flag-tagged E1 and E2. The results show a fast increase of total MeCP2 levels immediately after KCl treatment followed by a decrease to basal levels at around 4 h after treatment (Fig. 2f). Interestingly, we observe a completely different pattern between MeCP2 isoforms. As expected, E1 shows a trend similar to that of total MeCP2, rapid upregulation upon depolarization that is maintained during 3–4 h, and then, protein levels decrease to reach, in this case, approximately 50% of the initial E1 levels (Fig. 2f). By contrast, E2 shows a stable pattern, exhibiting levels similar to those of non-treated cells throughout the whole duration of the experiment (Fig. 2f). Hence, our data confirm the existence of different dynamics of MeCP2 isoforms that are consistent with a different role of the proteins within the neuronal context.

### Genome-wide distribution of MeCP2-E1 and MeCP2-E2 isoforms

**Fig. 3:**
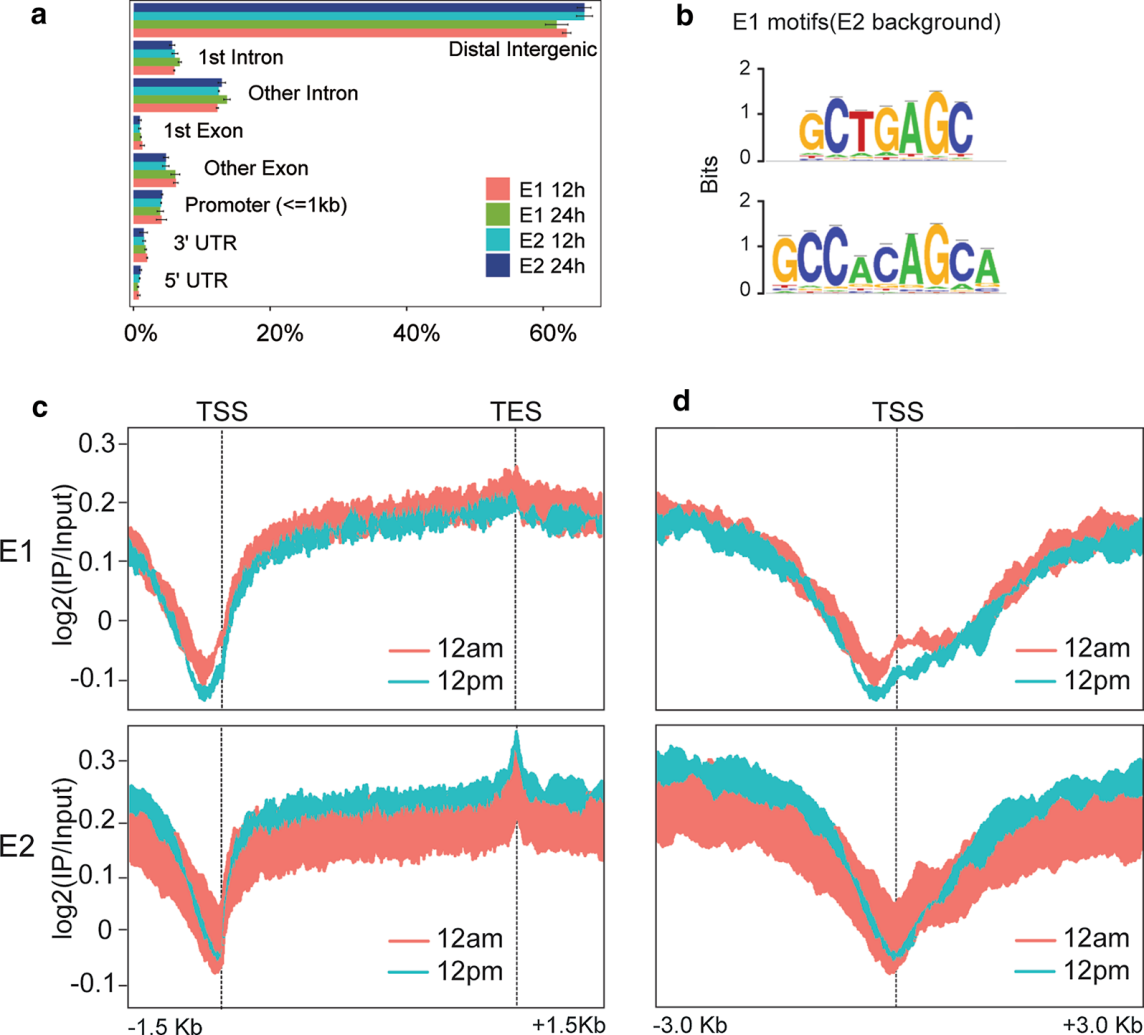
Genome-wide distribution and dynamics of MeCP2 isoforms. **a** Bar plot depicting the distributions of regions enriched in the MeCP2 isoforms across eight defined genomic features. **b** DNA-binding motifs enriched for MeCP2-E1 (and excluding E2) shown as motif logos based on aligned, over-represented patterns found in the binding sites. The overall height of each stack indicates the sequence conservation at that position (measured in bits), whereas the height of symbols within the stack reflects the relative frequency of the corresponding amino or nucleic acid at that position. **c** ChIP-seq average profiles across 3 Kb upstream the TSS and 3 Kb downstream the TES of genic regions occupied by E1 at 12 a.m. and 12 p.m. (top) and E2 at 12 a.m. and 12 p.m. (bottom). **d** Details of the previous representations focusing in a 6 Kb region surrounding the TSS of E1 and E2 bound genes (top and bottom panels, respectively)

The differences described between E1 and E2 in terms of their affinity for methylated DNA and dynamics might have an influence on and/or reflect a differential genomic distribution. Chromatin immunoprecipitation and sequencing (ChIP-seq) of E1 and E2 in frontal cortices of mice euthanized at 12 a.m. and 12 p.m. showed broad distributions for both isoforms, as previously noted for total MeCP2 (Additional file 3: Fig. S3A and B, [21–23]. The overall distribution of the isoforms along different genomic regions was similar (FDR ≤ 0.001, SICER algorithm: window 600 bp; gap 200 bp; Fig. 3a). Despite the similar isoform’s general distribution, we were able to identify different significantly enriched binding motifs specific for E1 (using E2 bound peaks as background), GCTGAGC (e-value: 1.2 e−145), and GCCACAGCA (e-value: 2 e−99) indicating a differential binding site preference (Fig. 3b). As MeCP2 has been described to be a transcriptional regulator, we analyzed the distribution of MeCP2 isoforms around transcribed regions. The average binding profiles of E1 and E2 to regions spanning 1.5 kb upstream of transcription start sites (TSS) to 1.5 kb downstream transcription end sites (TES) demonstrate a consistently similar binding pattern for both isoforms, with a marked depletion at TSS and a peak at TES (Fig. 3c).

Interestingly, a closer inspection around TSS regions (Fig. 3d) revealed a marked depletion of E2 precisely at the TSS. In contrast, the E1 isoform is depleted before the TSS and corresponding to the −1 nucleosome region, with a slight increase on the TSS at 12 a.m. that decreases at 12 p.m. These results suggest a differentiated role of the two isoforms in shaping the chromatin structure around the TSS.

**Fig. 4:**
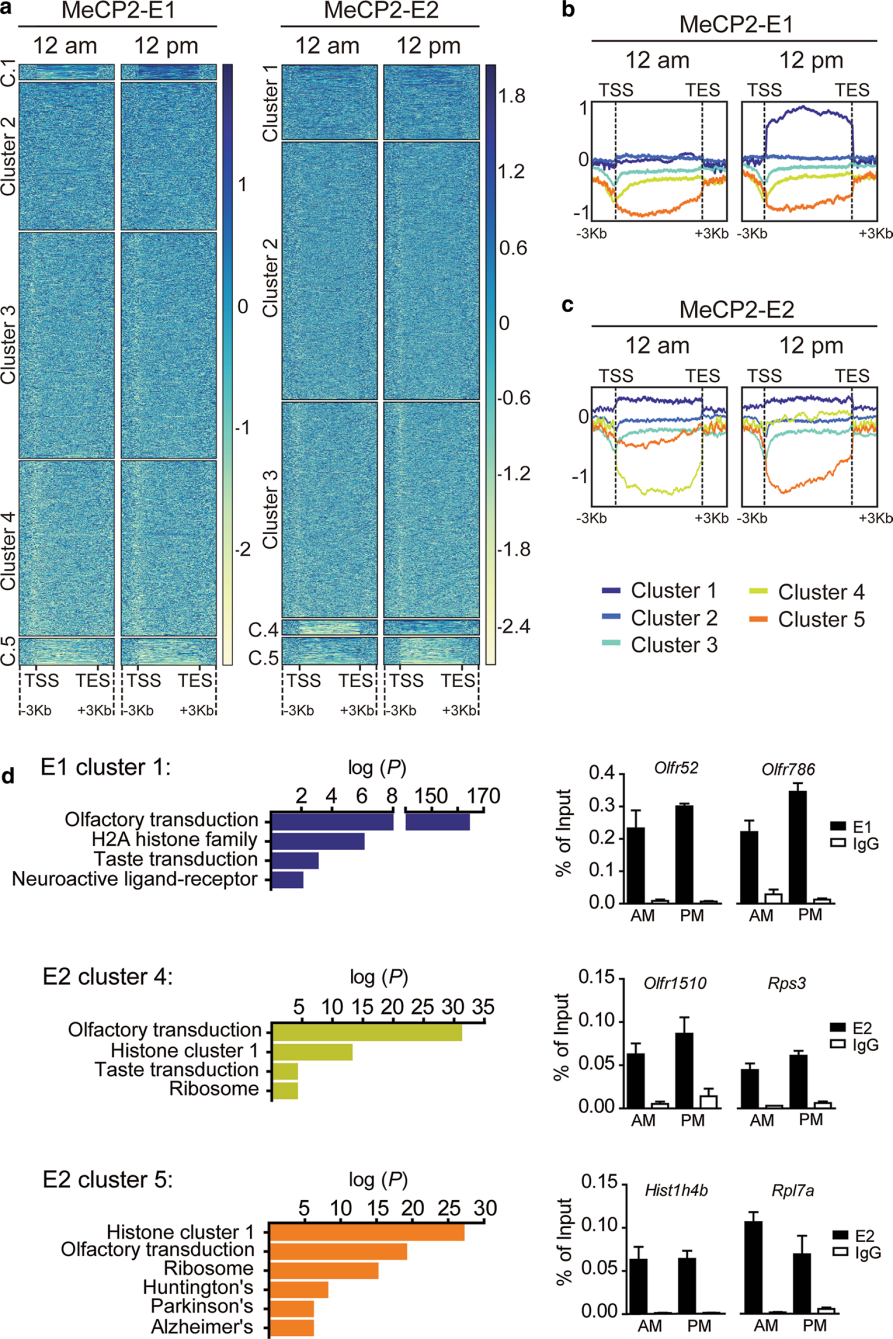
MeCP2-E1 and E2 isoforms display diurnal dynamic genomic binding. **a** Heatmaps representing the log2 ratios obtained for E1 and E2 ChIP experiments; each column is divided into five clusters using the k-means algorithm. Protein occupancy is represented by color intensity, where the darker the color, the higher the protein enrichment. **b** Comparison of E1 enrichment at 12 a.m. vs. 12 p.m. showing occupancy differences in different clusters of interest. **c** Heatmap depicting the E2 12 a.m. vs. 12 p.m. shows a dynamic binding in clusters 4 and 5 (yellow and orange, respectively). **d** Left graphs: top-enriched functional pathways (−log10 (*P*)) of genes included in dynamic E1 and E2 gene clusters [Kyoto encyclopedia of genes and genomes (KEGG)]. Right graphs: ChIP-qPCR results validating MeCP2-E1 and E2 variations in occupancy of genes included in each of the gene clusters obtained

We then clustered the genes based on their MeCP2 occupancy. Heatmap clusters and profiles for the log2 ratio plots failed to reveal any differential binding of the isoforms to specific gene clusters (data not shown); however, we detected daily differences for each isoform occupancy throughout gene bodies (Fig. 4a). For instance, E1 cluster 1 showed a flat profile at 12 a.m. and an increased binding at 12 p.m. (Figure 4b). In the case of E2, cluster 4 exhibited an increased binding at 12 p.m. compared to 12 a.m., while cluster 5 displayed lower binding at 12 p.m. (Figure 4c). Functional pathways associated with genes present in each cluster were analyzed using the Kyoto encyclopedia of genes and genomes (KEGG) (Fig. 4d left graphs). All three clusters were enriched in genes related to sensory transduction like olfaction or taste, and with histone proteins (E1 was associated with genes encoding H2A family members (*p* value: 1.16 e^−06^) and E2 mainly with members of the histone cluster 1 (cluster 4 *p* value: 1.65 e^−13^ and cluster 5 *p* value: 2.23 e^−27^). MeCP2 isoform-specific enrichments were related to neuroactive ligand–receptor interaction in E1 and ribosomal proteins in E2. Interestingly, cluster 5 contains several genes associated with the neurodegenerative diseases Huntington (*p* value: 4.17 e^−08^), Parkinson (*p* value: 9.87 e^−06^) and Alzheimer (*p* value: 9.15 e^−06^). ChIP-qPCR validations of randomly selected genes of each cluster confirmed the general trends observed in our ChIP-seq-analysis, despite the very slight variations of the isoforms’ occupancies during the day (Fig. 4d right graphs). Overall, our results suggest that beyond the common functions in which both isoforms are involved, they regulate different sets of genes and display distinct dynamics on their genomic occupancy, reinforcing the existence of non-overlapping roles.

### MeCP2-E1 and E2 protein partners

**Fig. 5:**
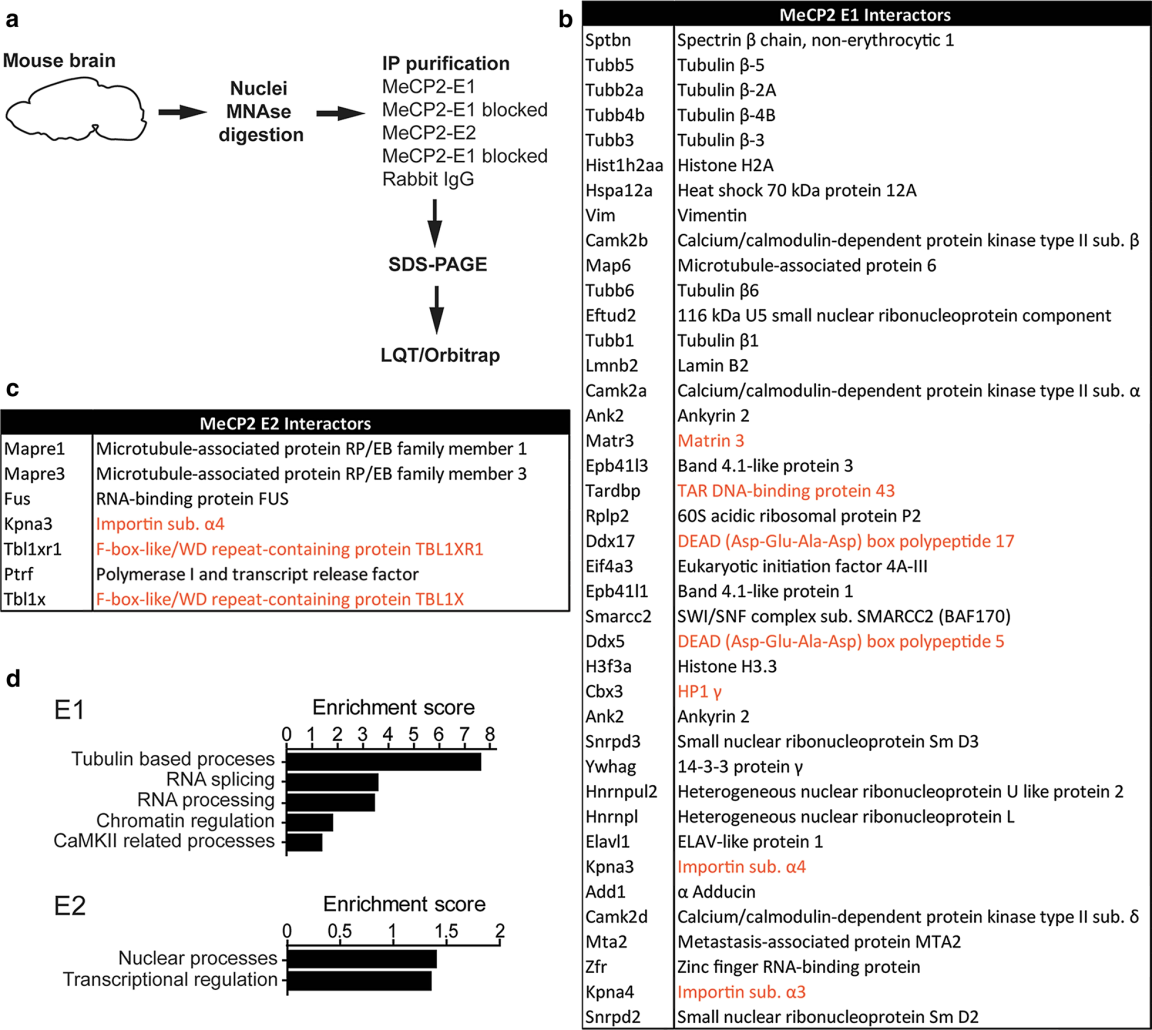
MeCP2-E1 and E2 interacting proteins. **a** Schematic workflow of the proteomic analysis. **b**, **c** Proteins partners identified for each isoform. **d** Selected pathways enriched in E1 and E2 interacting proteins as identified by functional clustering (DAVID Gene Ontology Bioinformatics Resources). Newly identified interactors are shown in black and previously identified interactors are highlighted in orange

IDP proteins are characterized by their inability to acquire a stable secondary structure when free in solution. This confers the structural flexibility that enables them to serve as scaffolds for the recruitment of partners and thus function as interacting hubs [24]. Interestingly, IDPs, including MeCP2, usually acquire ordered structures upon binding to their interacting partners, allowing the exposure of molecular recognition features (MoRFs) to further make contacts with other molecules [25, 26]. Thus, the possibility exists that the aforementioned E1 and E2 differences in unfolding temperature and affinity for DNA could expose differential interacting surfaces. These attributes together with their previously discussed expression patterns [3, 4, 27] raise the possibility that E1 and E2 might be involved in non-overlapping molecular functions that perhaps could be defined through the identification of their protein interactors. Therefore, we decided to perform a comprehensive proteomic analysis to look for MeCP2-E1 and E2 protein partners. Endogenous E1 and E2 from whole brain lysates were immunoprecipitated, using normal rabbit IgG and blocking of E1 and E2 antibodies with blocking peptides were used as negative controls. Co-immunoprecipitated proteins were separated by SDS-PAGE and different gel fractions sectioned for protein identification by mass spectrometric analysis (Fig. 5a).

We chose proteins identified by at least 2 significantly matching peptides which were absent from the negative controls. This filter rendered 40 interacting proteins for the E1 isoform and 7 for E2 (Fig. 5b, c). As a good validation for our approach, we detected several previously described MeCP2 interactors (Fig. 5b, c, interactors highlighted in orange [28–34]).

Functional clustering of co-eluted proteins (DAVID [35]) uncovered functional enrichments, especially for E1 (Fig. 5d). E1 co-eluted proteins are highly enriched for β-Tubulins, the building blocks of microtubules, and microtubule-associated proteins such Adducin 1 (Add1) or microtubule-associated protein 6 (Map6). Importantly, microtubule assembly initiates from the centrosome, organelle associated with MeCP2 function in microtubule stability, and mitotic spindle organization [36–38]. Proteins related to mRNA splicing and mRNA processing were also highly represented among E1 partners (for example, 116 kDa U5 small nuclear ribonucleoprotein component [Eftud2], Heterogeneous nuclear ribonucleoproteins L [Hnrnpl] or DEAD (Asp-Glu-Ala-Asp) box polypeptides 5 and 17 [Ddx5 and Ddx17]). MeCP2 functions on RNA splicing or mRNA processing have been previously described [33, 39, 40], but still lack deep investigation. As we expected, functions related to chromatin regulation are also enriched among MeCP2-E1 partners, as we found the nucleosome-core histone H2A and the variant H3.3, the chromatin regulators Brg1 associated factor 170 (BAF170), member of the switch/*sucrose* non-fermenting (SWI/SNF) complex, and MTA2, subunit of the nucleosome remodeling deacetylase (NuRD) complex [41]. Functional network analysis (STRINGv10 [42]) revealed a higher than expected number of connections between all E1 and E2 interactors (Additional file 4: Fig. S4; *p* value < 0.001), and suggests the participation of E2 in processes similar to those involving E1, but through the interaction with a different set of protein partners. In this regard, among E2 interactors, we found the microtubule-associated protein RP/EB family members 1 and 3 (Mapre1 and Mapre3), important for microtubule organization [43]. The E2 interactor fused in sarcoma (FUS) is involved in mRNA processing, with *Mecp2* being one of its known target genes [44]. In the chromatin regulation group, we found that E2 specifically interacts with two recently described MeCP2 protein partners: Transducin-β-like 1 (Tbl1) and Tbl1-related 1 (Tbl1r1), components of the nuclear receptor co-repressor (N-CoR) complex [28, 29]. Interestingly, E2 also interacts with the polymerase I transcription and release factor (Ptrf), and protein involved in ribosomal DNA (rDNA) transcription [45].

E1 co-eluted proteins include spectrin β1, lamin B2, the band 4.1 proteins B and N, and matrin 3 (the latter was previously reported to interact with E1 in neuronal nuclei [31]), components of the nuclear matrix [46], classically defined as a fibrogranular structure which consists of nucleoskeleton/nuclear lamina networks and associated proteins [47, 48]. Of note, one of the best characterized components of the nuclear matrix is the attached region-binding protein (ARBP), a chicken MeCP2 orthologue [49] that binds methylated DNA within matrix attachment region (MAR) elements [48, 50].

Overall, the lack of shared protein partners by the MeCP2-E1 and E2 isoforms suggests their involvement in similar general mechanisms like RNA processing, chromatin control of transcription, or microtubule regulation, but performing non-redundant functions through the interaction with different partners.

## Discussion

The existence of mutations affecting only the MeCP2-E1 isoform in Rett patients [e.g., p.Ala2Val; [13, 51]] suggest that endogenous E2 expression cannot compensate for the lack of functional E1. An important question thus arises as to whether this is simply related to the lower levels of E2 found in neurons [27] or it is due to the existence of E1-specific functions that cannot be provided by the E2 isoform. The different cellular distribution of the two isoforms and their distribution during brain development (Additional file 1: Fig. S1) also suggest a different functionality.

The NTD is the only structural feature that differs between the two MeCP2 isoforms, and currently, there is a lack of information regarding any potential functional difference between E1 and E2 NTDs. The NTD of these isoforms has generically been described as a highly disordered region able to acquire secondary structure, as demonstrated by the coil-to-helix transitions exhibited in the presence of hydrogen-bond stabilizers [26]. Such conformational transitions contribute to enhancing the MBD affinity for DNA [14]. To the best of our knowledge, the biophysical characterization of the interaction of NTD-MBD fragments of the two MeCP2 isoforms with DNA is the first of its kind (Fig. 1). Most of MeCP2 functions rely on its ability to bind nucleic acids, and in this regard, these results uncover a fundamental ninefold difference in affinity for DNA of E2 over E1 isoform. This could be one of the basic structural features responsible for shaping the functional discrepancies between the isoforms.

The main differences observed here between the MeCP2 isoforms can be summarized as follows: E1 (the major isoform of MeCP2 in neurons) shows a lower DNA-binding affinity and a lower structural stability (Fig. 1). E1 also exhibits a higher basal degradation rate in various neuronal settings and enhanced dynamic fluctuations of protein levels via diurnal rhythm oscillations and neuronal depolarization (Fig. 2). Within a neuronal context, these attributes are especially interesting given the peculiar chromatin relationship between MeCP2 and the linker histone H1 in this tissue. Under normal conditions, neurons express MeCP2 to near histone-octamer levels and contain half the amount of H1. By contrast, when MeCP2 is not expressed, H1 returns to the levels which are observed in other somatic tissues [52]. Therefore, it is likely that MeCP2-E1 could function within this setting as a DNA-methylation-dependent highly dynamic linker histone, needed to allow for rapid chromatin structural changes in response to external stimuli. This is particularly important in neurons, given their versatile ability to readily modify gene expression as a result of their unique methylome [53, 54].

In this regard, signal transduction from the cell surface to the genome often relies on the cytoskeleton–nucleoskeleton–chromatin interconnection [46]. Importantly, we have identified MeCP2-E1 and E2 protein partners associated with every part of this system: the cytoskeleton (i.e., tubulins, Map6, and Mapre1 and 3), nuclear envelope/matrix-associated proteins (Lamin B2, Band 4.1 proteins, Spectrin, or Matrin 3), and chromatin (histone proteins, Hp1γ, Mta2, or Baf170) (Fig. 5). Our findings open up the possibility of MeCP2 functioning as an important player in signal transduction. In particular, E1 could play a prominent role in the neuron-specific nucleoskeleton–chromatin connection, due to its remarkable abundance and the dynamism observed upon the application of external stimuli such as neuronal depolarization.

Because of its higher abundance, E1 appears to exhibit a more dynamic behavior; however, E2 also exhibits an oscillating genomic-binding nature. Surprisingly, most of the daily MeCP2 isoform-binding differences observed overlap with genes encoding for sensorial receptors, such as olfactory (ORs) and taste (TASRs) receptors (Fig. 4d). This seemingly counterintuitive result is very interesting as preliminary observations have shown expression of ORs and TASRs in brain regions not related to the direct detection of odors and flavors [55]. The study of the so-called “ectopic ORs” (outside olfactory epithelium) is in its infancy, but apparently, they act as chemoreceptors which are important to maintain cellular homeostasis, and some of them are able to activate complex cellular responses mediated by neurotransmitters or hormones [55]. The expression of olfactory receptors has been described to be upregulated in MeCP2 KO mice and downregulated in mice overexpressing the protein. These data support a potential role for MeCP2 in transcriptional regulation of these genes in different regions of the brain structures such as cerebellum, amygdala, and hypothalamus [21]. More importantly, the expression of these receptors in brain is altered in neurodegenerative diseases such as Parkinson’s, Alzheimer’s and in prefrontal cortex in schizophrenia [55], disorders in which MeCP2 expression has been also observed to be dysregulated [16]. Our ChIP-seq results reinforce this MeCP2 function and add a dynamic component to it.

Quite unexpectedly, all gene clusters displaying dynamic diurnal binding of the two MeCP2 isoforms were also enriched in gene-encoding replication-dependent (RD) histones. Expression of RD histones has been recently detected in terminally differentiated cells and tissues, including neurons and brain [56, 57]. Some of these genes encode histone isotypes with a certain sequence divergence [56], and could possibly affect the histone interactions within the nucleosome. Further analyses will be required to assess if MeCP2 has any regulatory effect on the expression of such genes, but it is tempting to speculate a possible role in the generation of variant nucleosomes present in adult brain [56].

An additional distinctive function for MeCP2-E2 which could be inferred from our results has to do with ribosomal gene expression regulation. Our co-immuno-precipitation experiment demonstrated the interaction of E2 with Ptrf (Fig. 5c). Transcription of ribosomal genes has been reported to be dependent on the DNA methylation status [58] and occurs through the formation of nucleotide loops linking initiation and termination gene regions, process in which Ptrf participation is essential [45]. Our ChIP-seq results provide evidence for a dynamic MeCP2-E2 genomic binding to ribosomal genes (Fig. 4). In support to our results, MeCP2 has been previously linked to nucleolar changes during neuronal maturation [59].

Another observation made through our ChIP-seq analysis is that the two isoforms possess significant differences in DNA-binding site preference; E1 targets are significantly enriched for the DNA motifs GCTGAGC and GCCACAGCA. Interestingly, the latter motif contains the trinucleotide CAC, described by Lagger and colleagues to be, when methylated, a high affinity-binding site for MeCP2 in brain [22].

## Conclusion

Overall, the present work provides support to the notion that Rett syndrome arises from the simultaneous impairment of different cellular functions involving both MeCP2 isoforms. For instance, mutations of E1 may have a larger impact in neuronal chromatin structure and stimuli-dependent gene expression dynamics. We have previously described in *MeCP2* KO mice a decrease in the circadian gene oscillations of the brain-derived neurotrophic factor (*Bdnf*) and somatostatin (*Sst*) genes [20]. By contrast, similar mutations in E2 could be involved in the deregulation of ribosomal expression or microtubule control.

The seemingly contradictory literature available to date regarding the degree of functional overlapping of MeCP2 isoforms is likely the result of the lack of studies carried out on the endogenous native proteins. Overall, our results provide strong support for the existence of both different and overlapping functions between the two. Importantly, our study uncovers some functional aspects of MeCP2 that were previously unknown. It opens the door to further investigation that will be helpful to understand the role of this complex protein in the healthy state and the consequences of its deregulation in Rett syndrome and other neurological disorders.

## Methods

### Animals and cell lines

The *Mecp2*-null mouse strain [60] was purchased from Jackson Laboratories (003890B6.129P2(C)-Mecp2 < tm1.1Bird >/J), maintained on a CD1 background, and genotyped as described in [61]. C57BL/6 mice and Sprague–Dawley rats were maintained under standard animal house conditions (12 h dark–light cycles on ad libitum food and water intake). Cortical primary neurons were prepared from Sprague–Dawley rat embryos as described previously [62]. For depolarization assay, neuronal activity was blocked by pre-treatment of the cells with 1 mM tetrodotoxin (TTX), 100 mM DL-2-amino-5-phosphonovalerate (APV), and 20 mM 6-cyano-7-nitroquinoxaline-2, 3-dione (CNQX). Depolarization was achieved by 30 min exposure to 55 mM KCl in Tyrode buffer. Transfections with plasmids expressing Flag-MeCP2 isoforms (kind gift from Angus Wilson [63]) were performed using the Amaxa rat neuron nucleofector kit (Lonza) following the manufacturer’s instructions.

Human cell lines SH-SY5Y and HEK 294 were obtained from the American Type Culture Collection (ATCC) and were maintained in DMEM supplemented with 10% FBS, penicillin–streptomycin and l-glutamine (Gibco) in a 37 °C, 5% CO_2_ humidified incubator. Differentiation was performed as previously described [64]. Cells were transfected with MeCP2-E1 and MeCP2-E2 C-terminal GFP [5] using Lipofectamine 3000 (Invitrogen). Cycloheximide (CHX) treatments were performed 48 h after transfection.

#### Western blot

Mouse brains (brain development) for each development time period were used to prepare western blot samples as per [65]. Nuclear extracts of HEK293 cells expressing the 3 × Flag versions of E1 and E2 were used to prepare serial dilutions and calculate MeCP2-E1/MeCP2-E2 ratios. Frozen frontal cortices (circadian) and cell pellets (CHX) were homogenized on Laemmli buffer 3% B-Mercaptoethanol. In all experiments, proteins were separated by sodium dodecyl sulfate-polyacrylamide gel electrophoresis (SDS-PAGE) [66] and transferred onto nitrocellulose membranes (GE Healthcare), and blocking by incubating in 5% skimmed milk in PBS with 0.1% Tween 20. Membranes were incubated with specific antibodies, either overnight at 4 °C or for 1 h at room temperature. Antibodies and dilutions used were as follows: MeCP2 1:5000, β-actin 1:20,000, Histone H4 1:20,000 Histone H3 1:20,000 (Abcam), Flag 1:5000 (Sigma-Aldrich), EGFP 1:1000 (Thermo Fisher), in house rabbit polyclonal antibodies MeCP2 E1 1:1000 and E2 1:500. Fluorescent secondary antibodies were (Li-Cor) 1:10,000. Densitometries were performed with Li-Cor Image Studio Light software.

#### Protein expression

Plasmids were transformed into BL21 (DE3) Star *E. coli* strain. Protein expression was induced with 1 mM isopropyl 1-thio-β-d-galactopyranoside (IPTG) at 18 °C overnight. Cells were sonicated and treated with benzonase (Merck-Millipore). Proteins were purified using immobilized metal ion affinity chromatography (IMAC) in a HiTrap TALON column (GE-Healthcare Life Sciences) with two washing steps before a 10–150 mM imidazole elution gradient. Removal of the histidine-tag was performed with GST-tagged PreScission Protease (GE-Healthcare). Further purification was performed using a HiTrap TALON column and a GST TALON column (GE-Healthcare). The identity of all proteins was checked by mass spectrometry (4800plus MALDI-TOF/MS, Thermo Fisher). Stability and binding assays were performed at different pH and buffer conditions [50 mM Tris (pH 7–9), 0–150 mM NaCl; 50 mM Pipes (pH 7); 50 mM Phosphate (pH 7)]. Buffer exchanges were performed using a 10 kDa-pore size ultrafiltration device (Amicon centrifugal filter, Merck-Millipore) at 4000 rpm and 4 °C.

#### Double-stranded DNA

HPLC-purified single-stranded DNA fragments were purchased from Integrated DNA Technologies and annealed. Sequences correspond to the promoter IV of the mouse brain-derived neurotrophic factor (BDNF) [15].

#### Fluorescence spectroscopy

Thermal unfolding studies were performed in a Cary Eclipse fluorescence spectrophotometer (Varian—Agilent) in three steps. Fluorescence emission spectra were recorded from 300 to 400 nm (excitation 290 nm and bandwidth 5 nm). Protein concentration was set at 5 µM. Thermal stability assays were performed at a heating rate of 1 °C/min and at the wavelength for maximal spectral change (330 nm). Thermal unfolding experiments were analyzed considering a two-state unfolding model [15]. The stabilizing effect upon dsDNA interaction was assessed performing thermal denaturations in the presence of methylated and unmethylated DNA (at 10 µM) under the same conditions.

#### Isothermal titration calorimetry (ITC)

Proteins–dsDNA interactions were characterized using an Auto-iTC200 microcalorimeter (MicroCal—Malvern Instruments). 3–5 µM protein was titrated with 50 µM dsDNA (2 µL titrant every 150 s). Interaction parameters were obtained as previously described [15]. Buffer-independent binding parameters and protons released were determined by calorimetric titrations using buffers with different ionization enthalpies [15], and binding heat capacity was determined by calorimetric titrations at different temperatures.

#### Fluorescence recovery after photobleaching (FRAP)

MeCP2-E1 and MeCP2-E2 isoforms were expressed in HEK293T cells in chambered cover glass culture plates (Nunc™; NalgeNunc). Experiments were performed at 37 °C and 5% CO_2_. Confocal time-lapse images of frames (512 × 512 pixels) were captured at 488 nm excitation with 0.05 transmissions for GFP-tagged protein post-bleach recovery. Images were recorded with a minimum of 10 pre-bleach frames, 250 µs bleach time with 405 nm laser line at 100% transmission, and 150 post-bleach frames were recorded at equal time intervals.

#### Cycloheximide-chase assay

Cells were treated with 10 µg/mL of cycloheximide (Sigma-Aldrich) and harvested at the indicated times. Samples collected at each time point were then analyzed by western blot.

#### Mass spectrometry to determine MeCP2 PTMs

All protein samples were digested overnight at 37 °C with trypsin, using 50:1 protein:enzyme ratio. Digested peptide mixtures were desalted using C_18_ reverse phase columns, and then loaded onto a 50 cm × 75 μm ID column with RSLC 2 μm C_18_ packing material (EASY-Spray, Thermo-Fisher) with an integrated emitter, and then eluted into a Q-Exactive™ Hybrid Quadrupole-Orbitrap™ mass spectrometer (Thermo-Fisher) using an Easy-Spray nLC 1000 chromatography system (Thermo-Fisher). The mass spectrometer was operated with 1 mass spectrometry (MS) spectrum followed by 10 MS/MS spectra in a data-dependent mode. The MS was acquired with a resolution of 70,000 FWHM (full width at half maximum), a target of 1 × 10^6^ ions, and a maximum scan time of 120 ms. Using a relative collision energy of 27%, the MS/MS scans were acquired with a resolution of 17,500 FWHM, a target of 1 × 10^6^ ions, and a maximum scan time of 120 ms. A dynamic exclusion time of 15 s was used for the MS/MS scans. XCalibur 2.2 (Thermo-Fisher Scientific) was used to acquire the raw data files and further processed with the PEAKS 7 search engine (Bioinformatics Solutions) using a database consisting of the wild-type MeCP2 constructs. Ascores were assigned for the peptides and PTMs using the PEAKS 7 software.

#### Chromatin immunoprecipitation

Mice were euthanized 6 and 18 h after first light stimulus (12 a.m. and 12 p.m., respectively). One frontal cortex was crosslinked in 1x PBS 0.5% formaldehyde at room temperature for 5 min and quenched by adding 0.125 M Glycine for 5 min. The tissue was centrifuge and washed twice with ice-cold PBS. The pellet was resuspended in 1 mL ChIP lysis buffer [10 mM HEPES (pH7.9), 1.5 M MgCl_2_, 10 mM KCl, 0.5 mM DTT, and 0.1% NP-40], dounce-homogenized (10 strokes), and incubated on ice for 20 min. Nuclei were centrifuged at 2500×*g* for 5 min and resuspended in 300 µL of RIPA buffer [50 mM Tris–HCl (pH 8), 150 mM NaCl, 0.5% SDS, 0.5% Sodium deoxycholate, 1% Triton-100]. Suspension was sonicated in a Bioruptor (Diagenode) at high power for 15 min with 30 s on/off intervals. Chromatin was centrifuged at 16,000×*g* for 10 min. Supernatant was diluted ten times with RIPA buffer without SDS and pre-cleared for 2 h with 20 µL of Dynabeads Protein G magnetic beads (Thermo Fisher). Eight microns of MeCP2 E1 and E2 antibodies (produced in house, Additional file 2: Fig. S2B) and Normal Rabbit IgG (Cell Signaling Technologies) were bound to 25 µL of Dynabeads Protein G following the manufacturer’s instructions and incubated 1 h in PBS/5% BSA. Inmunoprecipitations were performed at 4 °C overnight while tumbling. Supernatants were discarded and beads washed twice with 1 mL of low salt buffer [50 mM Tris–HCl (pH 8.0), 150 mM NaCl, 0.1% SDS, 1% NP-40, 1 mM EDTA, 0.5% Sodium deoxycolate], twice with 1 mL of high salt buffer [50 mM Tris–HCl (pH 8.0), 500 mM NaCl, 0.1% SDS, 1% NP-40, 1 mM EDTA, 0.5% Sodium deoxycolate], twice with 1 mL of LiCl buffer [50 mM Tris–HCl (pH 8.0), 250 mM LiCl, 0.1% SDS, 1% NP-40, 1 mM EDTA, 0.5% sodium deoxycholate], and twice with 1 mL of TE. Chromatin was eluted in elution buffer (100 mM NaHCO_3_, 1% SDS) and reversal of the crosslinking was carried out for 5 h at 65 °C. The samples were then incubated with proteinase K for 1 h at 65 °C and RNase A for 30 min at 42 °C. DNA was purified with a PCR purification kit (Qiagen).

#### Bioinformatics analysis of ChIP-Seq data

ChIP-seq libraries were pooled and sequenced paired-end 75 on a HiSeq 2500 (Illumina). We detected binding sites using SICER (window 600 bp, gap 200 bp) [67], and detected differential binding sites (e.g., E1 vs E2) with the same tool. Peaks were called in each sample independently. Increased and decreased peaks in the two replicates were then combined to provide reliable peaks, using MSPC (10^−4^ and 10^−8^ thresholds on *p* values defining stringent and weak peaks, respectively [68]. Location of the peaks was performed with ChipSeeker [69] on mm10 annotation. For visualization purposes, ChIP signal has been normalized to input signal using the signal extraction scaling (SES) method [70]. RSAT peak-motif tool [71] was used detect over-represented motifs in the detected peaks. For cluster generation, we merged replicates and computed the fold-change distribution along the gene body, the TSS, and the TES. We then computed the fold-change distribution along each gene and clustered the genes into five groups using the k-means method. Pathway enrichment analysis was performed with WebGestalt [72].

#### qPCR

Each PCR was carried out in triplicate using SYBR Green PCR Master Mix (Applied Biosystems) and following the manufacturer’s instructions. Fluorescent signals were acquired by the Stratagene Mx3005P qPCR System (Agilent Technologies), and primer sequences upon request.

#### Co-immunoprecipitation and mass spectrometry

One whole brain/immunoprecipitation was processed as per [73] with slight modifications. MNase digestion was performed during 30 min at 37 °C with 150 units MNase (Worthington). After centrifugation, supernatant was kept on ice and reaction was stopped by addition of EDTA (10 mM final concentration). The remaining pellet was resuspended in digestion buffer with 2 mM CaCl_2_, and 50 ud MNase were added and incubated for 20 min at 37 °C. The reaction was stopped by addition of EDTA (10 mM final concentration), the sample was centrifuged and supernatant kept. Supernatant was pre-cleared for 1 h with 50 µL of Dynabeads Protein G magnetic beads (Thermo Fisher). Negative controls for the IP were obtained by blocking E1 and E2 paratopes with E1 and E2 specific peptides [E1: CAAAAPSGGGGGGEEER; E2: MVAGMLGLREEKC (New England Peptides)]. Antibodies were blocked during 45 min by tumbling at room temperature with a fivefold excess of the specific peptide. 10 µg of each antibody [E1, E1 blocked, E2, E2 blocked and normal rabbit IgG (Cell Signaling Technologies)] were bound to 50 µl of magnetic beads according to the manufacturer’s instructions. Immunoprecipitations were carried out overnight at 4 °C while tumbling. Antibody–protein complexes were washed and proteins eluted with 2x SDS buffer, boiled for 10 min and proteins were run on SDS-PAGE. The gel was stained with Coomassie blue and different sections were excised for subsequent analysis. Mass spectrometry was then performed using a nano-HPLC system (Easy-nLC II, Thermo Fisher), coupled to the ESI source of an LTQ Orbitrap Velos (Thermo Fisher), using conditions described previously [74]. MS data were acquired using a data-dependent method. The data acquisition also utilized dynamic exclusion, with an exclusion window of 10 ppm and exclusion duration of 10 s. MS events used 60,000 resolution FTMS scans, and MS/MS events used ITMS scans, with a scan range of m/z 400–2000 in the MS scan. MS/MS data were analyzed using Mascot. The data were compared to the Uniprot Mouse database, using trypsin digestion with up to three missed cleavages, a peptide tolerance of 5 ppm, and MS/MS tolerance of 0.3 Da. Acetylated N-termini and oxidation of methionine were included as variable modifications.

## Supplementary information

**Additional file 1: Fig. S1.** (A) Western blot analysis of the changes in the level of expression of MeCP2-E1 and E2 isoforms during brain development (*n* = 6). (B) Summary of thermal unfolding results for NTD-MBD E1 and NTD-MBD E2 performed in different conditions (pH 7, pH8 and pH9 and at pH7 in presence of methylated dsDNA or unmethylated dsDNA). (C) Fluorescence recovery after photobleaching (FRAP) to determine diffusion and binding kinetics of human wild type MeCP2-E1 and E2 in HEK293T. (1) Real time post-bleach recovery of GFP tagged isoforms (bleaching 1000 ms/frame for 2 frames). Red lightening indicates bleach spots. (2) and (3) Comparative illustration of average half maximal recovery time and mobile fraction of wild type MeCP2-W1 and E2, respectively. Mean and S.E. are shown. (4) FRAP recovery curves normalized to 1, showing chromocenter recovery in 151 frames.

**Additional file 2: Fig. S2**. A (1) Western blot showing the levels of total MeCP2 and MeCP2 isoforms upon SH-SY5Y differentiation. (2) Expression changes of general (Sox2) and neuronal (Syt1, Syn1, Tubb3) differentiation markers as detected by Reverse Transcriptase quantitative Polymerase Chain Reaction (RT qPCR). Data represent mean ± S.E.M (*n* = 3). (B) (1) Western blot showing untransfected HEK 293 cells and HEK 293 cells expressing 3xFlag-MeCP2 E1 and 3xFlag-MeCP2 E2. Immunoblots were performed using the following antibodies: panMeCP2, MeCP2-E1 and MeCP2-E2 (Three upper panel). Equal loadings were assessed by Coomasie blue gel staining (lower panel). (2) Western blot performed after immunoprecipitation of endogenous MeCP2-E1 and MeCP2-E2 from whole brain lysates. Normal rabbit IgG was used as negative control. Immunoprecipitated proteins were run in 10% gels to differentiate isoforms sizes and panMeCP2 antibody was used for staining. (3) Ponceau staining showing equal loadings of wild type (WT) and Adrian Bird’s knock out (KO) mouse (60) brain samples. Samples were run on 12% SDS PAGE (66). (4) Western blots of Total MeCP2, MeCP2-E1 and MeCP2-E2 antibodies. H4 was used as a normalizer.

**Additional file 3: Fig. S3.** (A) Summary table showing the amount of ChIP seq mapped reads per sample and number of reads post filtering. (B) Integrative genomics viewer (IGV) (23) profiles of E1 and E2 ChIP-seq based on sequence reads of each sample. Data represent a 20 Mb region of chromosome 17. Numbers in the right of each track represent track heights.

**Additional file 4: Fig.** **S4**. MeCP2 isoforms functional networks as determined by using STRINGv10 software.

## Data Availability

Data sets generated and/or analyzed during the current study have been deposited to the Gene Expression Omnibus Database (GEO) under the accession number GSE130277.

## Abbreviations

MeCP2: methyl CpG-binding protein 2
NTD: N-terminal domain
MBD: methyl-binding domain
ChIP: chromatin immunoprecipitation
dsDNA: double-stranded DNA
ITC: isothermal titration calorimetry
FRAP: fluorescence recovery after photobleaching
IDP: intrinsically disordered protein
NM: N-terminal methionine
NME: N-terminal methionine excision
PTM: post-translational modification
CHX: cycloheximide
WB: western blot
EGFP: enhanced green fluorescent protein
DIV: days in vitro
KEGG: Kyoto encyclopedia of genes and genomes
TSS: transcriptional start site
TES: transcriptional end site
qPCR: quantitative polymerase chain reaction
MoRF: molecular recognition features
SDS-PAGE: sodium dodecyl sulfate–polyacrylamide gel electrophoresis
MAR: matrix attachment region
OR: olfactory receptor
TASR: taste receptor
