# Supplementary figures and images for: MeCP2-E1 isoform is a dynamically expressed, weakly DNA-bound protein with different protein and DNA interactions compared to MeCP2-E2
Source: Epigenetics Chromatin. 2019 Oct 10;12:63. doi: 10.1186/s13072-019-0298-1 (PMC6786283; doi:10.1186/s13072-019-0298-1)

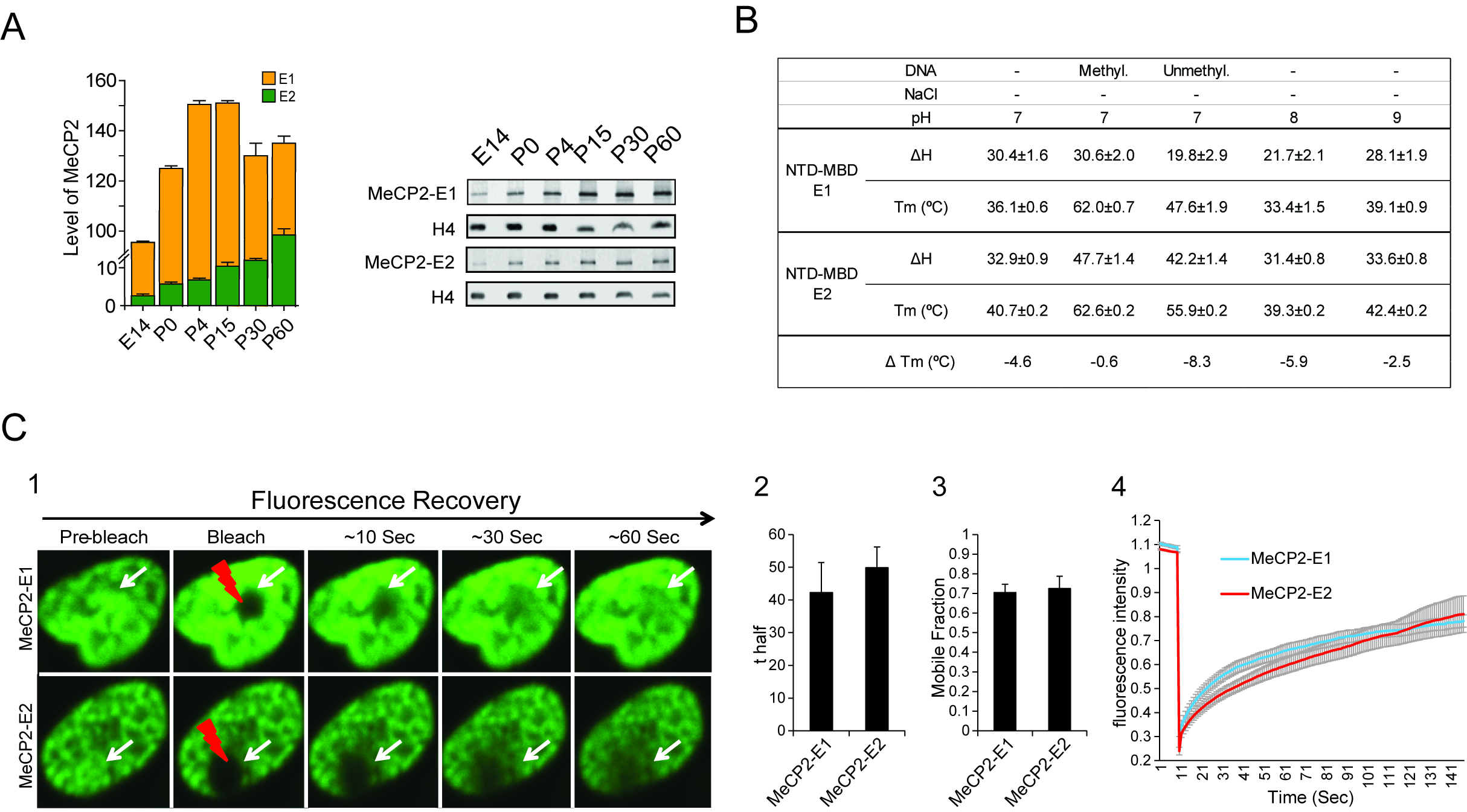

Supplement: Supplementary file 1 — Additional file 1: Fig. S1. (A) Western blot analysis of the changes in the level of expression of MeCP2-E1 and E2 isoforms during brain development (n = 6). (B) Summary of thermal unfolding results for NTD-MBD E1 and NTD-MBD E2 performed in different conditions (pH 7, pH8 and pH9 and at pH7 in presence of methylated dsDNA or unmethylated dsDNA). (C) Fluorescence recovery after photobleaching (FRAP) to determine diffusion and binding kinetics of human wild type MeCP2-E1 and E2 in HEK293T. (1) Real time post-bleach recovery of GFP tagged isoforms (bleaching 1000 ms/frame for 2 frames). Red lightening indicates bleach spots. (2) and (3) Comparative illustration of average half maximal recovery time and mobile fraction of wild type MeCP2-W1 and E2, respectively. Mean and S.E. are shown. (4) FRAP recovery curves normalized to 1, showing chromocenter recovery in 151 frames. [file 13072_2019_298_MOESM1_ESM.tif]

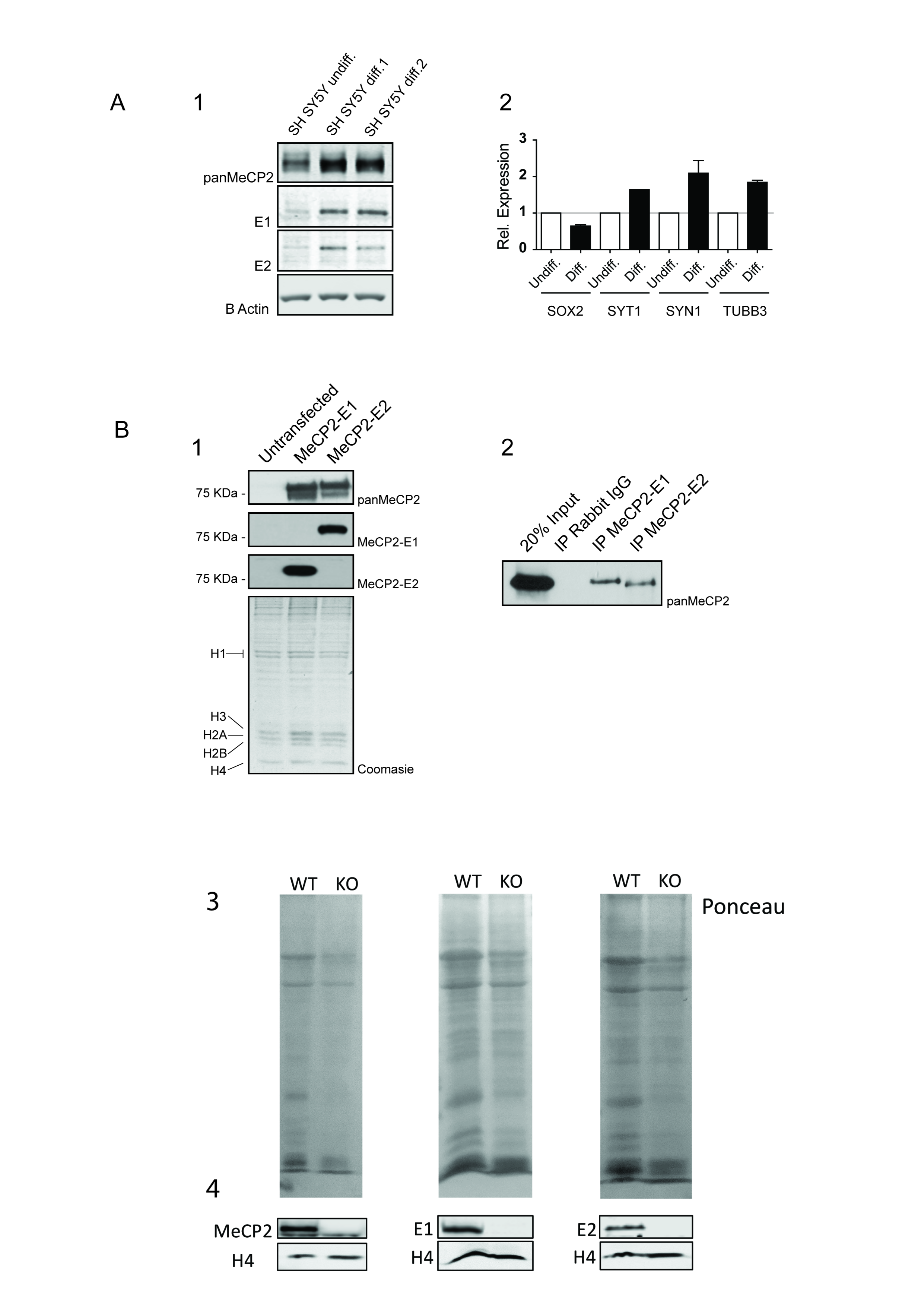

Supplement: Supplementary file 2 — Additional file 2: Fig. S2. A (1) Western blot showing the levels of total MeCP2 and MeCP2 isoforms upon SH-SY5Y differentiation. (2) Expression changes of general (Sox2) and neuronal (Syt1, Syn1, Tubb3) differentiation markers as detected by Reverse Transcriptase quantitative Polymerase Chain Reaction (RT qPCR). Data represent mean ± S.E.M (n = 3). (B) (1) Western blot showing untransfected HEK 293 cells and HEK 293 cells expressing 3xFlag-MeCP2 E1 and 3xFlag-MeCP2 E2. Immunoblots were performed using the following antibodies: panMeCP2, MeCP2-E1 and MeCP2-E2 (Three upper panel). Equal loadings were assessed by Coomasie blue gel staining (lower panel). (2) Western blot performed after immunoprecipitation of endogenous MeCP2-E1 and MeCP2-E2 from whole brain lysates. Normal rabbit IgG was used as negative control. Immunoprecipitated proteins were run in 10% gels to differentiate isoforms sizes and panMeCP2 antibody was used for staining. (3) Ponceau staining showing equal loadings of wild type (WT) and Adrian Bird’s knock out (KO) mouse (60) brain samples. Samples were run on 12% SDS PAGE (66). (4) Western blots of Total MeCP2, MeCP2-E1 and MeCP2-E2 antibodies. H4 was used as a normalizer. [file 13072_2019_298_MOESM2_ESM.tif]

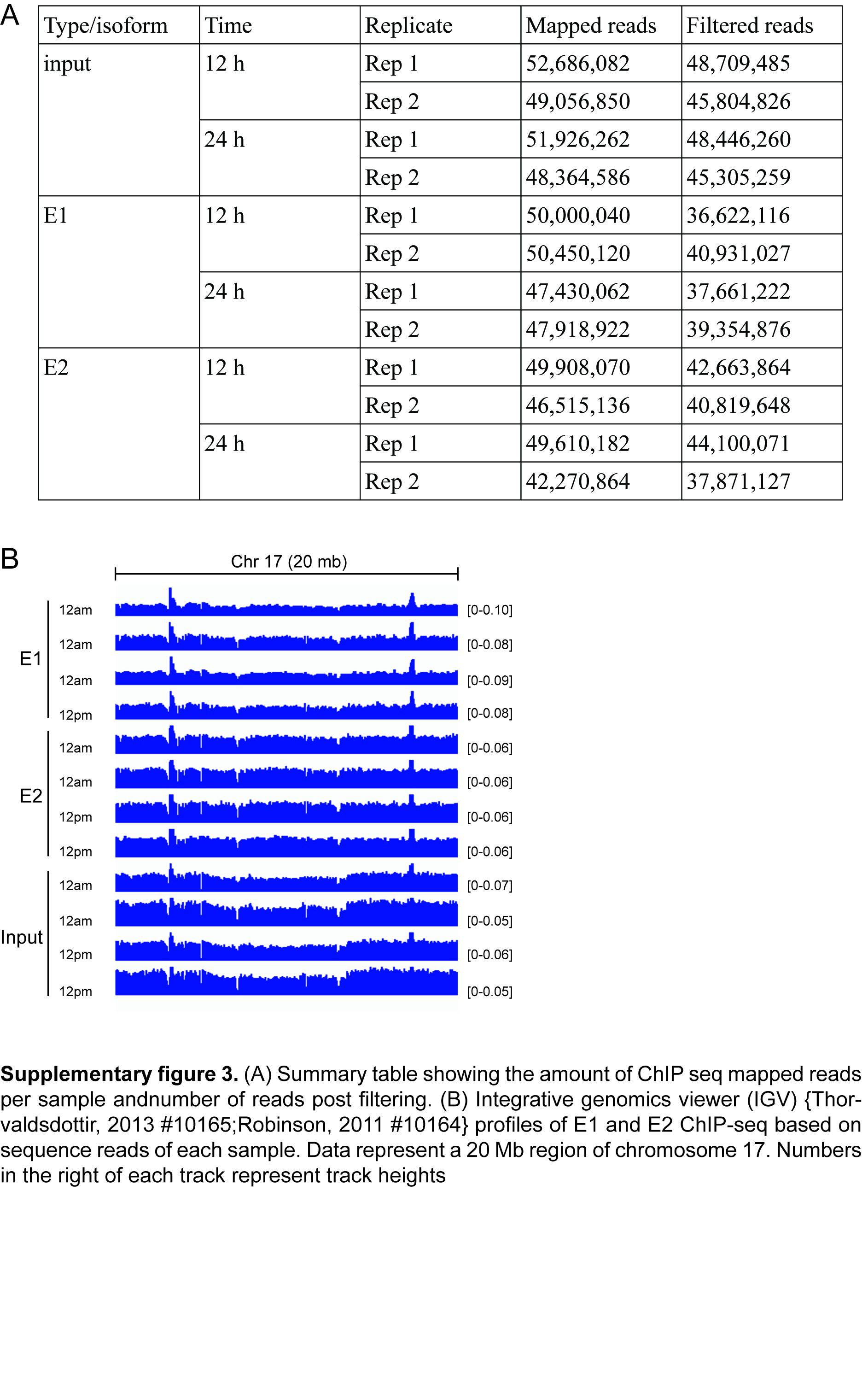

Supplement: Supplementary file 3 — Additional file 3: Fig. S3. (A) Summary table showing the amount of ChIP seq mapped reads per sample and number of reads post filtering. (B) Integrative genomics viewer (IGV) (23) profiles of E1 and E2 ChIP-seq based on sequence reads of each sample. Data represent a 20 Mb region of chromosome 17. Numbers in the right of each track represent track heights. [file 13072_2019_298_MOESM3_ESM.tif]

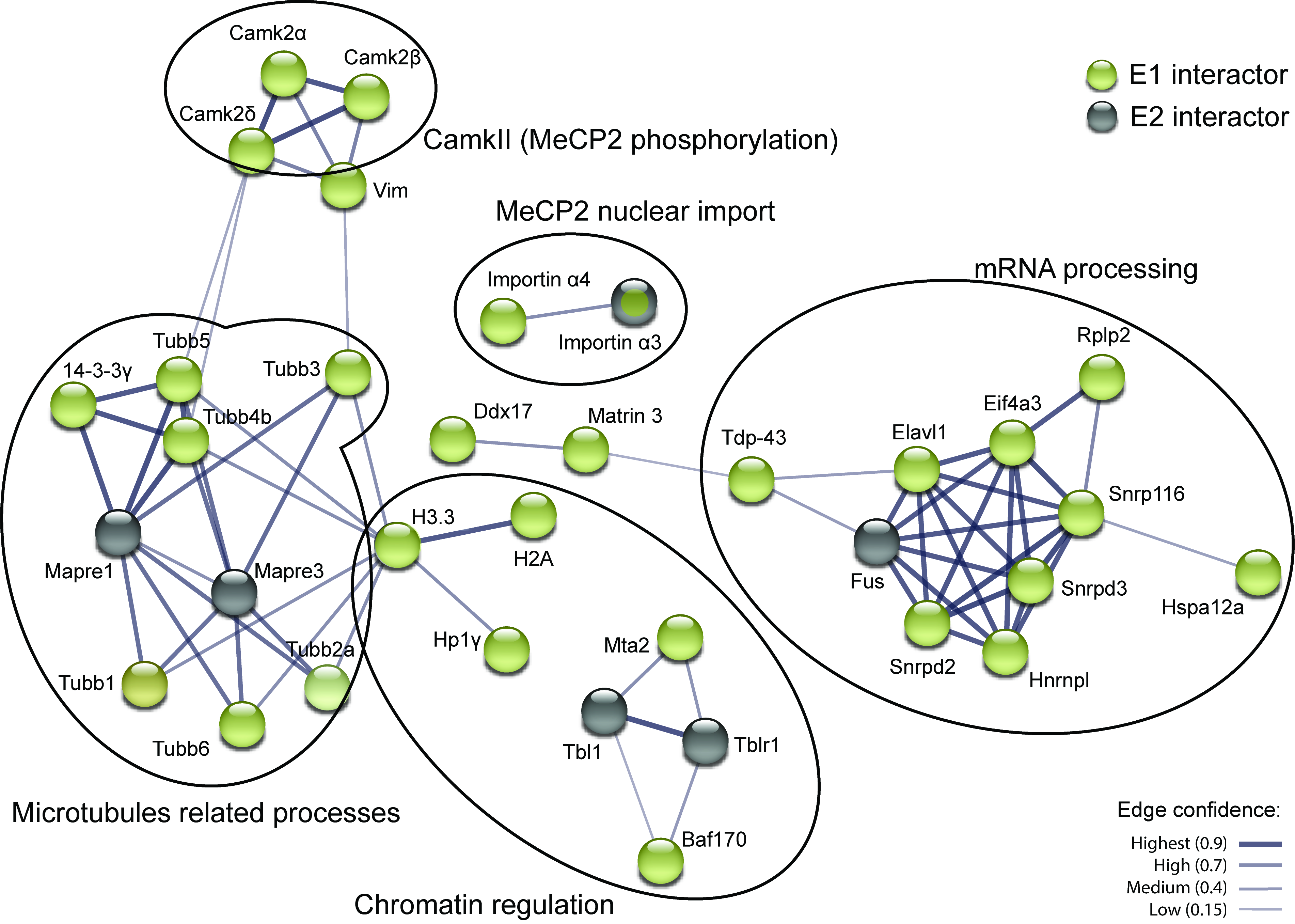

Supplement: Supplementary file 4 — Additional file 4: Fig. S4. MeCP2 isoforms functional networks as determined by using STRINGv10 software. [file 13072_2019_298_MOESM4_ESM.tif]
